# Cardiac renewing: interstitial Cajal-like cells nurse cardiomyocyte progenitors in epicardial stem cell niches

**DOI:** 10.1111/j.1582-4934.2009.00758.x

**Published:** 2009-04-20

**Authors:** L M Popescu, Mihaela Gherghiceanu, C G Manole, Maria Simonetta Faussone-Pellegrini

**Affiliations:** a Department of Cellular and Molecular Medicine, ‘Carol Davila’ University of Medicine and Pharmacy, Bucharest, Romania; b ‘Victor Babes’ National Institute of Pathology, Bucharest, Romania; c Department of Anatomy, Histology and Forensic Medicine, Section of Histology, University of Florence, Florence, Italy

**Keywords:** epicardium, subepicardium, interstitial Cajal-like cells, cardiomyocytes progenitors, cardiac repair, cardiac regeneration, myocardial remodelling, cardiac stem cells niches, shed microvesicle, epicardium-derived progenitor cells (EPDCs)

## Abstract

Recent studies suggested that various cell lineages exist within the subepicardium and we supposed that this area could host cardiac stem cell niches **(CSCNs)**. Using transmission electron microscopy, we have found at least 10 types of cells coexisting in the subepicardium of normal adult mice: adipocytes, fibroblasts, Schwann cells and nerve fibres, isolated smooth muscle cells, mast cells, macrophages, lymphocytes, interstitial Cajal-like cells **(ICLCs)** and cardiomyocytes progenitors **(CMPs)**. The latter cells, sited in the area of origin of coronary arteries and aorta, showed typical features of either very immature or developing cardiomyocytes. Some of these cells were connected to each other to form columns surrounded by a basal lamina and embedded in a cellular network made by ICLCs. Complex intercellular communication occurs between the ICLCs and CMPs through electron-dense nanostructures or through shed vesicles. *We provide here for the first time the ultrastructural description of **CSCN** in the adult mice myocardium, mainly containing **ICLCs** and **CMPs***. The existence of resident CMPs in different developmental stages proves that cardiac renewing is a continuous process. We suggest that ICLCs might act as supporting nurse cells of the cardiac niches and may be responsible for activation, commitment and migration of the stem cells out of the niches. Briefly, not only resident cardiac stem cells but also ICLCs regulate myocyte turnover and contribute to both cardiac cellular homeostasis and endogenous repair/remodelling after injuries.

## Introduction

The epicardium appears to be more than a simple serosal ‘umbrella’ over myocardium and this finding could bring out the ultimate gift left in Pandora's box – the hope – the possibility that epicardial progenitor cells could be persuaded to efficiently regenerate the damaged heart.

It is supposed, based on colourful, spectacular (*but low resolving power*) images using immunohistochemistry and/or confocal microscopy that epicardium is a source of both myocardial and supporting cells capable to replace cardiomyocytes and vessels in the damaged heart [1–17]. It has also been reported that allogeneic stimulated mesenchymal stem cells improve the ejection fraction [18], therefore stressing the importance of the specific supporting connective tissue.

Cellular therapies propose exogenous adult or embryonic stem cells [19–35] and endogenous cardiac stem cells [36–47] for repairing or replacing the damaged cardiac working cells but they were partially effective mainly due to the lack of a structural and functional integration with the pre-existing ‘working’ myocardium [48–52].

We have shown previously the existence of interstitial Cajal-like cells (ICLCs) as a distinct type of cell in human and mammalian myocardium and suggested that they play a role in supporting the cardiomyocytes three-dimensional organization and could influence (control) their microenvironment, *e.g*. paracrine secretion and/or shedding microvesicles [53–59]. Moreover, very recently, using cell culture and frozen sections, we highlighted ICLCs by fluorescence microscopy in the adult mice epicardium [60]. Although, canonical intestinal interstitial Cajal cells (ICC) are very well known [61–66], Faussone-Pellegrini's group identified ICLCs in human intestine beyond the classical ICC and suggested a supporting role for these cells in the three-dimensional organization and microenvironment of the enteric muscle coat [67].

Noteworthy, recent studies [68–69] described selected areas of the heart as functional units supposed to be cardiac stem cell niches (CSCNs) and now is (generally) accepted that adult stem cells reside in specialized niches that coordinate tissue renewal [69–73]. The cells of the niches seem to be connected to the pre-existing cardiomyocytes and interstitial supporting cells [68, 69] but the identity of the last (but not the least) cells is still unknown.

We report here for the first time the ultrastructural identification and description of the cardiomyocyte progenitors (CMPs) and their structural connections with the ICLCs as supporting nurse cells in epicardial stem cell niches in adult mice. Thus, we show unequivocally the existence of CSCN in adult epicardium and that ICLCs are not simple bystanders, but key actors in adult myocardial renewing. These findings would have important clinical implications for the management of cardiac diseases.

## Materials and methods

Hearts from four 16-week-old normal mice (two females and two males; B6129PF2/J strain) purchased from Jackson Laboratories (Bar Harbor ME, USA) were examined by transmission electron microscopy (TEM). The institutional ethical committee approved the study. Small fragments from atrial and ventricular myocardium with epicardium were processed according to routine procedures, as we previously described [55]. Light microscopy was performed on 1 μm semi-thin section stained with 1% toluidine blue and digital images were recorded using a CCD Axiocam HRc Zeiss camera with AxioVision software (Carl Zeiss Imaging solution GmbH, Germany), on Nikon Eclipse E600 microscope (Nikon Instruments, Inc., Tokyo, Japan). Ultrathin sections stained with uranyl acetate and Reynolds's lead citrate solutions were examined using a Morgagni 286 TEM (FEI Company, Eindhoven, The Nederlands) at 60 kV. Digital electron micrographs were recorded with a MegaView III CCD using iTEM-SIS software (Olympus, Soft Imaging System GmbH, Germany). Digital coloured TEM images were processed using Adobe Photoshop software (Adobe Systems, Inc., San Jose, CA, USA). The colour codes used to emphasize different types of cells are blue for ICLCs, brown for cardiomyocyte and progenitors and green for nerve fibres.

## Results

### Light microscopy

Semi-thin toluidine blue stained sections of the atrium and ventricle of adult mouse heart allowed to identify several cell populations in the subepicardium (i.e.the connective tissue in between the epicardial epithelium and the myocardial tissue). The subepicardium covering the atrium and ventricle appears as thin collagen tissue with scattered cellularity (Fig. 1A, B). Some of these cells were easily recognizable, such as mast cells, adipocytes, macrophages, lymphocytes and nerves, and were present in small numbers everywhere beneath the epicardium (Fig. 1). Cells with long processes are constantly present under the atrial and ventricular epicardium (Fig. 1A, B); however, they are not certainly recognizable as ICLCs and fibroblasts.

**1:**
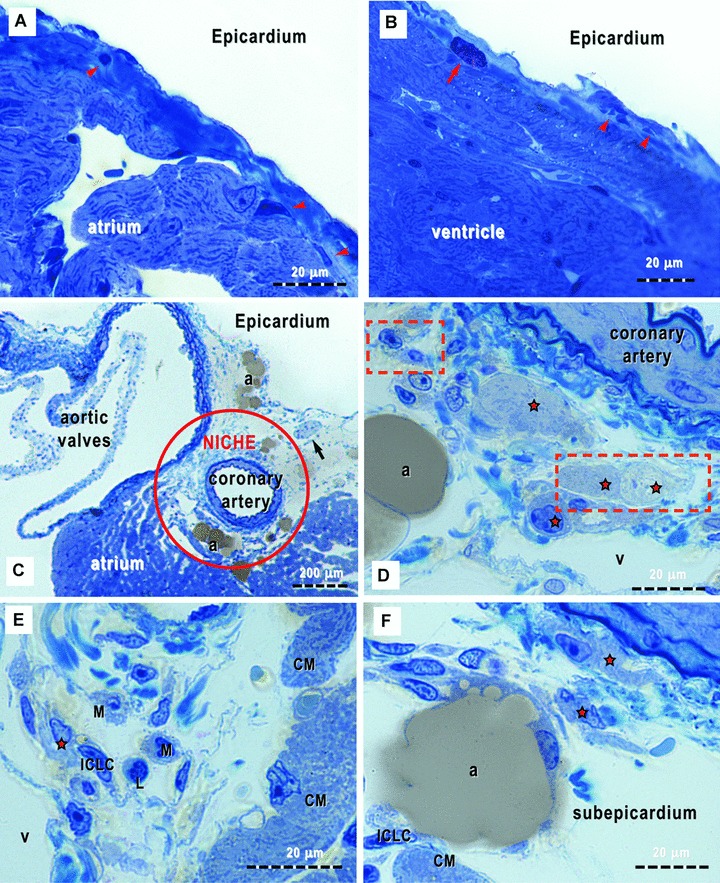
(**A**)-(**F**). Light microscopy of toluidine-blue stained semi-thin sections (∼1 μm, or less) of Epon-embedded epicardium from 16-week-old mice (supposed to be the equivalent of 25 human years). The loose connective tissue with very few cells (arrowheads) may be observed immediately below the epicardial mesothelium (the so-called subepicardium), in both cases of atrium (**A**) and ventricle (**B**). A metachromatically stained mast cell (red arrow) can be easily recognized. (**C**) shows the richness of cell population types surrounding the coronary artery and a cross sectioned nerve (arrow). Red circle marks the epicardial stem cell niche. (**D**)-(**F**) demonstrate different cellular silhouettes scattered in the loose connective tissue of the cardiac stem cell niche. Note, the presence of ‘young’ cardiomyocytes (red stars); the orange rectangles areas correspond to high-resolution electron microscope images in Fig. 6. ICLC – interstitial Cajal-like cells; CM – cardiomyocytes; L – lymphocyte; M – macrophage; v – venules and a – adipocytes.

In the subepicardial area at the origin of coronary arteries and aorta, the connective tissue is loose and particularly rich in cells (Fig. 1C). All types of cells already mentioned were present in this location, as well as cells we identified as belonging to the myocardial lineage (Fig. 1D–F) and we presumed to be CMPs in a cardiac niche.

### Transmission electron microscopy

Under TEM, all the afore-mentioned cells were identified as well as ICLCs (Figs. 2, 3A, inset, 4A, 6–9) and fibroblasts (Fig. 3B). Free smooth muscle cells were occasionally seen, all of them similar to the periarteriolar (data not shown). Some mononuclear cells showed unspecific ultrastructural appearance and they could be stem cells or cells under dormant state (data not shown). Moreover, the presumed CMPs (Figs. 4–7) were unequivocally identified under TEM. Peculiar relationships between the CMPs and the ICLCs were also seen giving rise to structures we identified as epicardial stem cell niche (Figs. 8–14).

**2:**
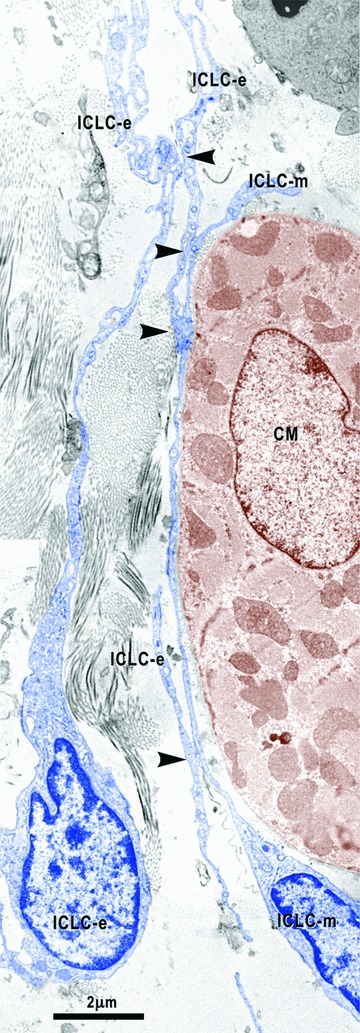
Digitally coloured image shows the close relationships between the epicardial ICLC (ICLC-e) and the ICLC bordering the myocardium (ICLC-m). Arrowheads indicate close contacts between epicardial ICLC and myocardial ICLC. ICLC – interstitial Cajal-like cells are blue coloured and CM – cardiomyocyte coloured in brown.

**3:**
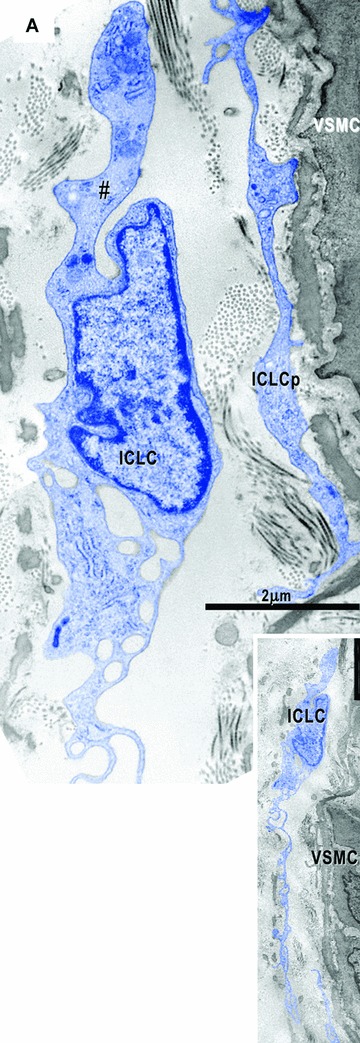

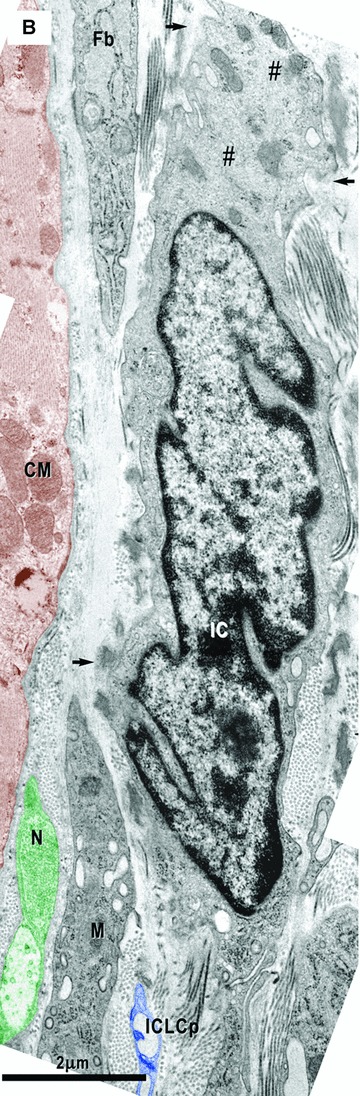
Electron micrographs show interstitial cells in the subepicardial area next to the coronary artery. (**A**) – Interstitial Cajal-like cells (ICLC, blue) have long and thin processes (inset), a lacunar cytoplasm, few cisternae of the rough endoplasmic reticulum and intermediate filaments (#). No basal lamina appears surrounding these cells. Another ICLC process (ICLCp) is near coronary smooth muscle cells (VSMC). (**B**) – Next to the atrial myocardium, an interstitial cell (IC) particularly rich in filaments (#) shows focal basal lamina-like material (arrows). N – nerve (green); M – macrophage; Fb – fibroblast; CM – cardiomyocyte (brown).

**4:**
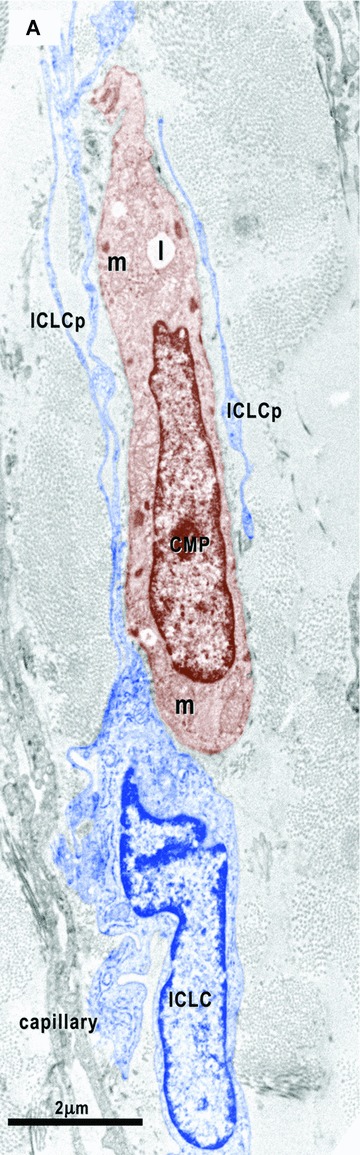

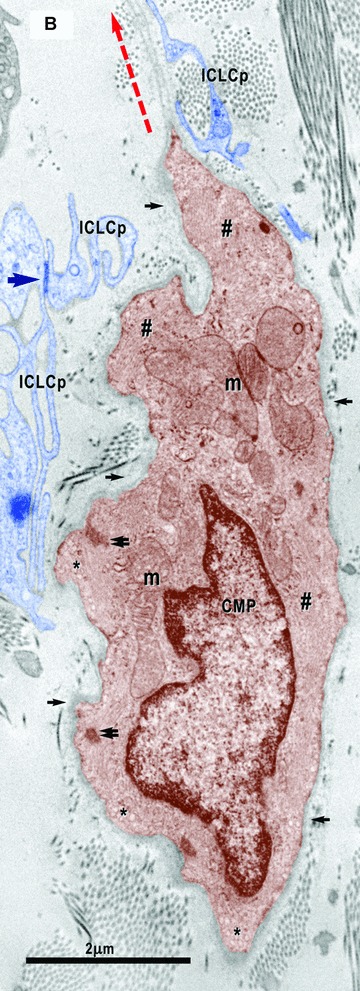

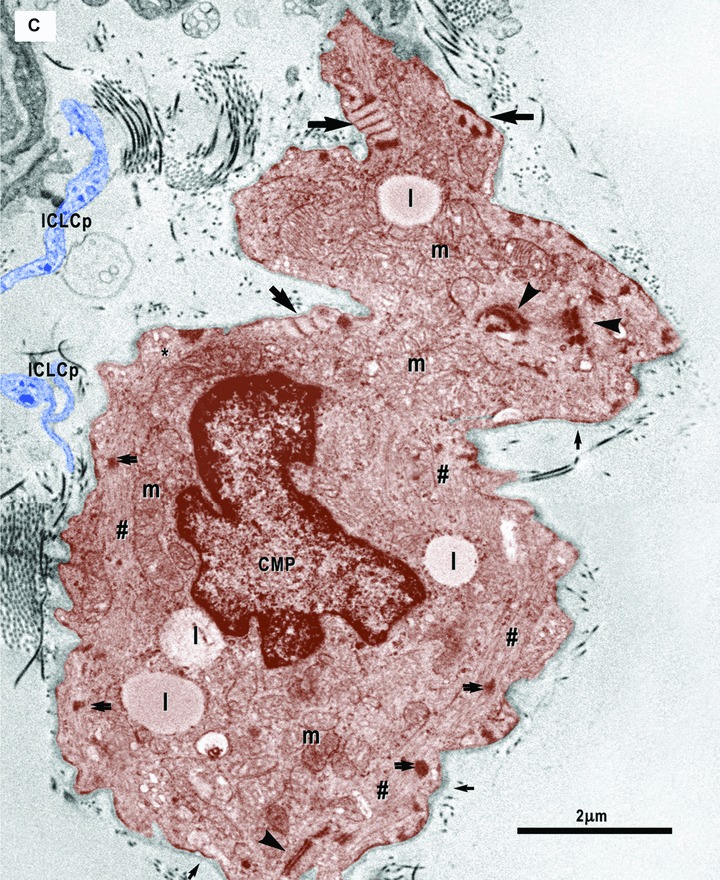
(**A**)–(**C**). Digitally coloured electron micrographs show cardiomyocyte progenitors (CMP, brown) in different stages of development and ICLC (blue). CMP have features of immature cardiomyocytes: a high nucleo-cytoplasmatic ratio and a continuous basal lamina (small arrows), numerous mitochondria (m) and ribosomes, few endoplasmic reticulum cisternae, some lipid droplets (l) and caveolae (*). These cells contain unorganized bundles of filaments (#) attached to electron dense structures (double arrows) similar to Z bands. (**A**) – ICLC processes (ICLCp) closely accompany the immature cardiomyocyte. **B**– The basal lamina exceeds the cellular profile and delimitates a slender acellular space. An ICLC process seems to support and direct this lamina (red dotted arrow). The large arrow indicates a gap junction in between two ICLCp. (**C**) – Note the presence of the subplasmalemmal leptofibrils (large arrows) and intracellular desmosome-like structures (arrowheads).

**6:**
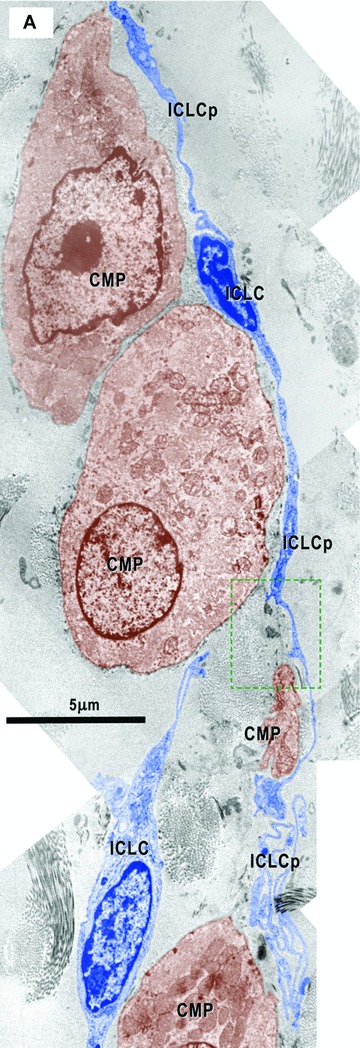

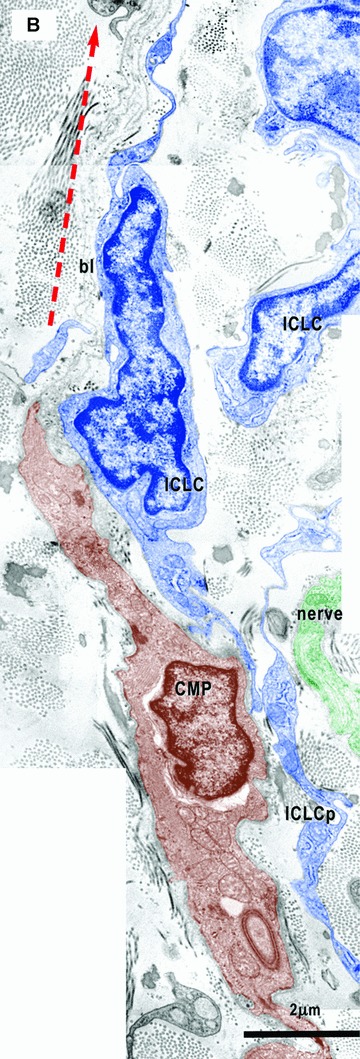
(**A**) and (**B**) Electron micrographs (from the areas marked in Fig. 1D) illustrate the relationships of the ICLC (blue coloured) with cardiomyocyte progenitors (CMP, brown). The ICLC processes (ICLCp) run parallel with the main axis of the CMP and seem to establish their direction of development. In (**B**) it is clear that the ICLC sustain a direction (red dotted arrow) for the basal lamina (bl) which goes beyond the CMP. Details from the upper area in Fig. 10C. The square mark area from A is enlarged in Fig. 10D.

**7:**
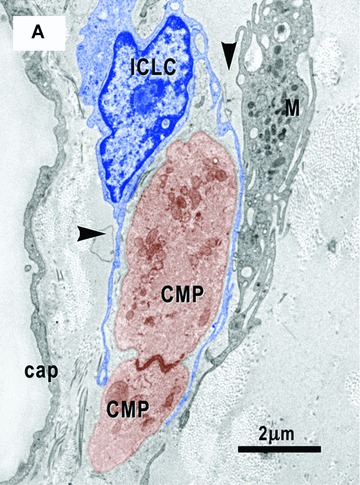

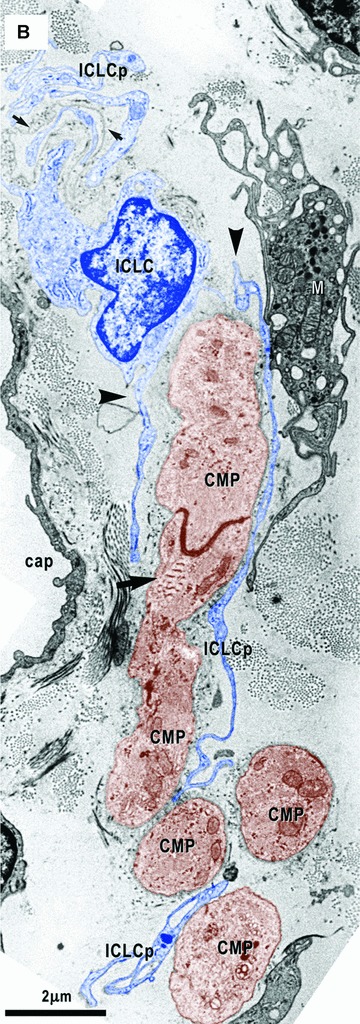
(**A**) and (**B**) Electron micrographs on serial sections show an ICLC (blue coloured) with two processes (arrowheads) which hold two cardiomyocyte progenitors (CMP, brown). Note close contacts of one ICLC process (ICLCp) with a macrophage (M). (**B**) – A basal lamina – like material surrounds an ICLC process (small arrows in the upper left corner). Leptomeric fibrils (arrow) connect the primitive intercalated disk with the plasma membrane. Cap – capillary.

**8:**
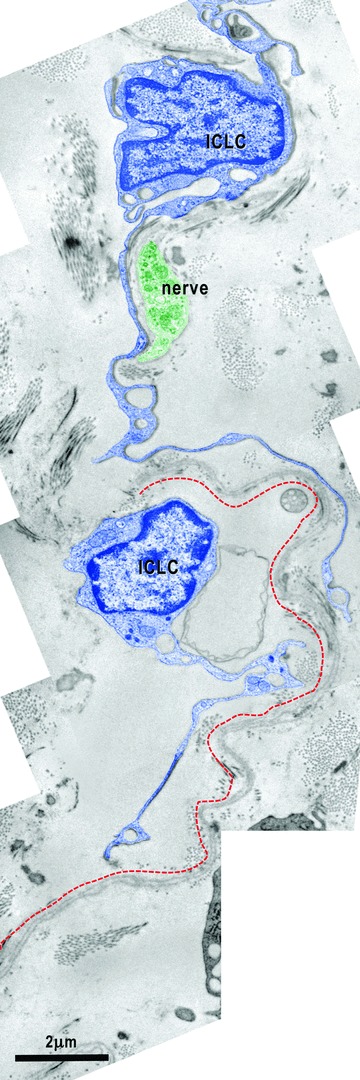
Electron micrograph illustrates the relationships of the ICLC (blue coloured) with a nerve fibre (green) and the basal lamina material that extends over the bodies of the cardiomyocyte progenitors. The processes of two ICLC guide a 30-μm-long bi-layered basal lamina (red dotted line).

**9:**
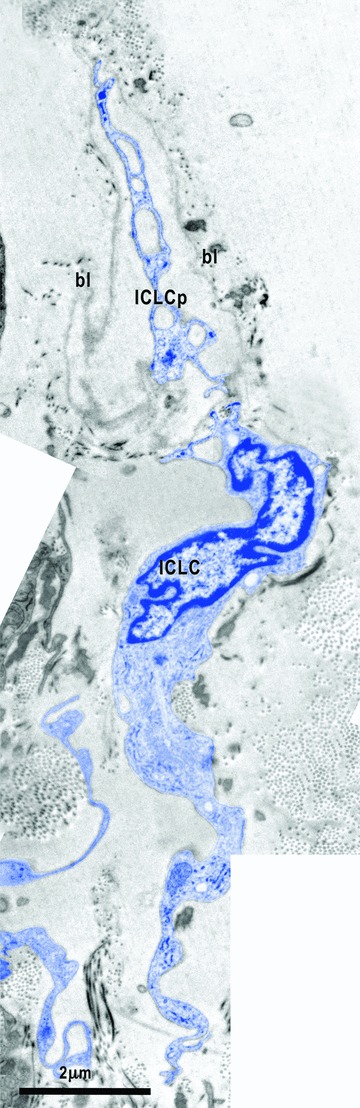
Electron micrograph illustrates the relationships of one ICLC (blue) with the basal lamina (bl). In this micrograph, one ICLC process (ICLCp) with a lacunar cytoplasm seems to be embedded in the space delimited by a continuous basal lamina.

**5:**
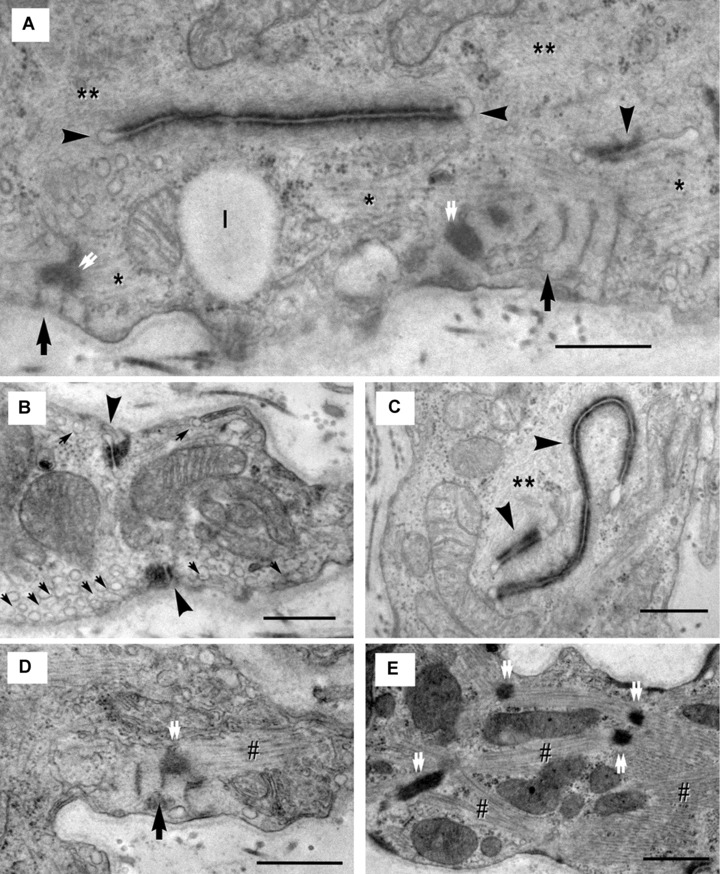
(**A**)-(**E**). Electron micrographs show ultrastructural features of the cardiomyocyte progenitors. (**A**) – Desmosome-like structures (arrowheads) are entirely located inside the cell and seem to organize thin filaments (**). Thick filaments (*) appear to be organized by leptofibrils (arrows). Z-like dense structures (double arrows) are visible at the end of leptofibrils. Image in (**B**) shows numerous caveolae (small arrows) and confluent intracytoplasmatic vesicles (primordial T tubules) and suggests that the desmosome-like structures (arrowheads) start from periphery and extend towards the centre of the cell. In (**C**), it is obvious that thin filaments (**) are organized by desmosome-like structures (arrowheads). (**D**) – Myofibrils (#) appear to be directed by leptofibrils (arrow) that anchor them to the plasma membrane throughout Z-like dense structures (double arrow). In (**E**), the nascent sarcomeres are visible: Z-like dense structures (double arrows) organize myofibrils (#) in an immature sarcomeric configuration. l – lipid droplet. Scale bar **=** 0.5 μm.

**10:**
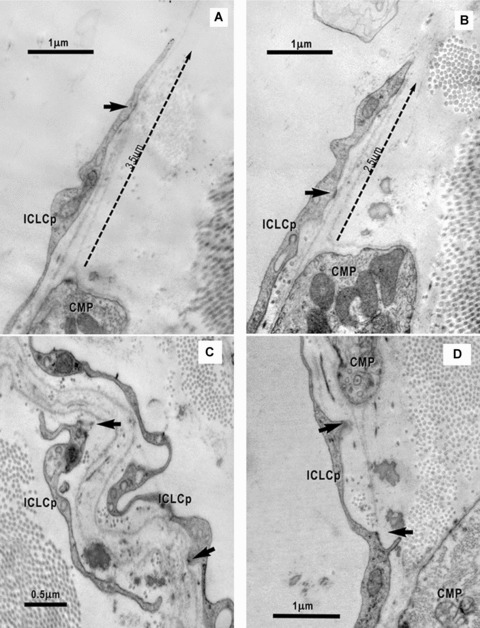
(**A**) and (**B**). Electron micrographs on serial ultrathin sections highlight the connections of the ICLC processes (ICLCp) with the basal lamina of the CMP by electron dense nanostructures (arrows). The ICLC process seems to support and guide the basal lamina, which exceeds the cellular profile of the CMP (dotted arrows). (**C**), (**D**) – High magnifications of the areas marked in Fig. 6 point out the close relationship of the ICLCp with the basal lamina of the CMP. Arrows indicate adjoining points.

**11:**
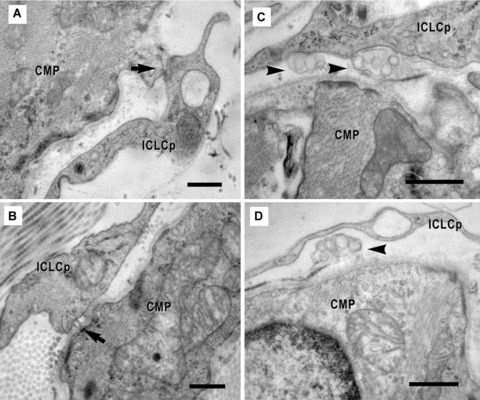
(**A**)-(**D**). Electron micrographs show the physical contacts (arrows) of the ICLC processes (ICLCp) with cardiomyocyte progenitors. The connections between the two types of cells could be: direct (**A**), through dense structures (**B**) or mediated by shed vesicles (arrowheads) (**C**, **D**). Scale bar **=** 0.5 μm.

**12:**
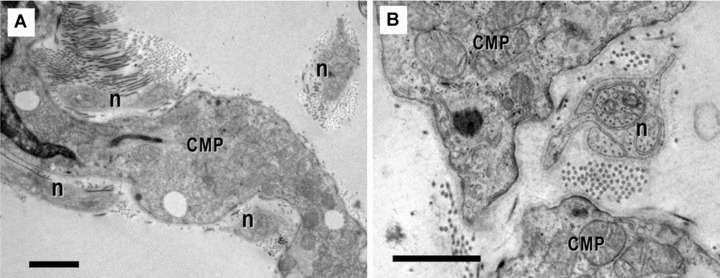
(**A**) and (**B**). Electron micrographs show the close vicinity of nerve fibres (n) with cardiomyocytes progenitors (CMP). Scale bar **=** 1 μm.

**13:**
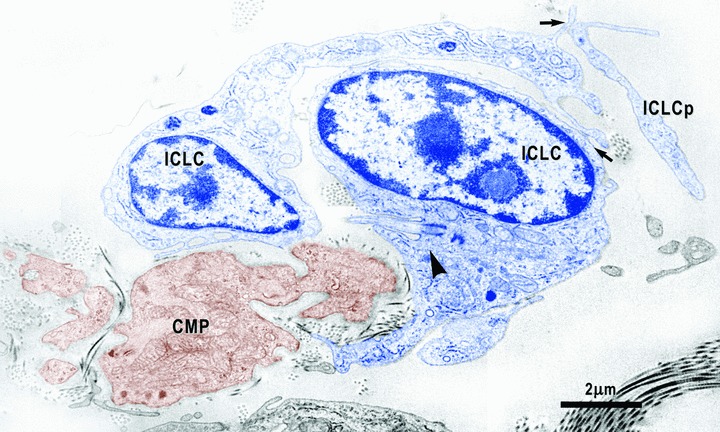
Electron micrograph shows close relationships between cardiomyocyte progenitors (CMP, brown) and ICLC (blue). One of the two ICLC has a cilium (arrowhead) directed towards the CMP. Arrows indicate close contacts between ICLC.

**14:**
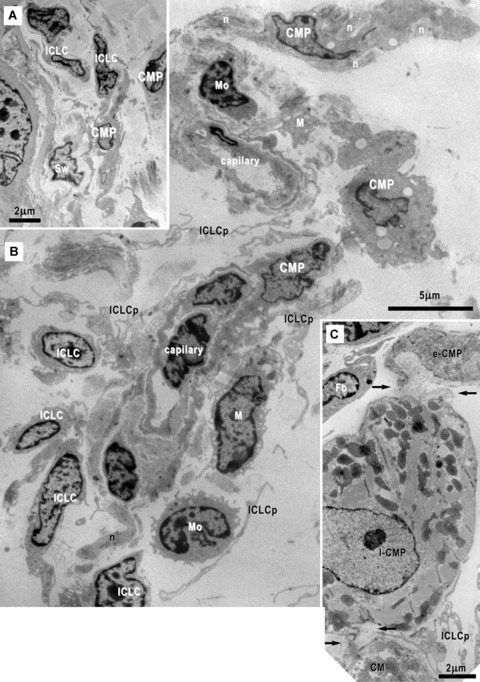
Electron micrographs of the cardiac stem cells niche in the subepicardium surrounding the coronary artery (**A**, **B**) and next to the peripheral adult cardiomyocytes (**C**). (**B**) Cardiomyocyte progenitors (CMP), interstitial Cajal-like cells (ICLC) and their inconspicuous processes (ICLCp), macrophages (M), mononuclear cells (Mo) and nerves (n) are clustered around capillaries. (**C**) Early (e-CMP) and late (l-CMP) cardiomyocyte progenitors (CMP) could be seen nearby an adult cardiomyocyte (CM). A basal lamina envelops these cells (arrows) closely assisted by ICLCp. Fb- fibroblast.

### Subepicardial interstitial Cajal-like cells

The cells identified as ICLCs fulfil ultrastructural criteria already established for this cell type [54, 55]:

1 Characteristic long (several tens of μm), thin and moniliform cell processes (Figs. 2, 3A inset, 4A, 6–9);
2 Cell-to-cell junctions (Figs. 2, 4B, 6);
3 Caveolae and coated vesicles (Figs. 10, 11);
4 Mitochondria, relatively well developed smooth and rough endoplasmic reticulum (Figs. 2, 6–9);
5 Intermediate (Fig. 3A) and thin filaments, microtubules and undetectable thick filaments;
6 A single cilium (Fig. 13) and
7 No basal lamina (Figs. 2–4, 6–11).

In the entire subepicardium, the ICLCs are located under the epicardial cells and around blood vessels. Similar to the atria, ventricle and myocardial sleeves [54–57], these ICLCs interconnect with each other through point cell-to-cell contacts or gap junctions (Fig. 4B), thus forming a three-dimensional network. Attachment plaques connect the ICLCs to the extracellular matrix and the perivascular elastic material (Fig. 3A) or to the basal lamina of the CMP (Fig. 10). Moreover, close contacts exist between the subepicardial ICLCs and the ICLCs bordering the myocardium (Fig. 2). The subepicardial ICLCs accompany all the blood vessels forming an incomplete sheath around them and the ICLC processes surround the cross-sectioned coronary artery wall (data not shown). In addition, the ICLCs showed a close vicinity with nerves (Figs. 6B, 8), blood capillaries (Figs. 4A, 7) and macrophages (Figs. 3B, 7).

### Subepicardial cardiomyocyte progenitors

All the cells identifiable as belonging to the myocardial lineage and reasonably considered to be CMPs have an oval or elongated shape with an irregular contour. CMPs are extremely variable in size (2–10 μm diameter and 15–30 μm length) but smaller than adult cardiomyocytes. The CMPs seem to be randomly oriented, without a preferential localization with the respect to the myocardium. Some slender CMPs were located around the coronary artery in between the ICLCs, a few CMPs with high degree of maturation were located in between coronary artery and the epicardium and some CMPs were located nearby the terminal myocardium but with very few connections with adult tissue (Fig. 14). All the CMPs presented immature structural features but they were in different stages of development: low (Figs. 3B, 4A, 5A), medium (Figs. 4B, C, 5B–D, 6B, 14) and high (Figs. 5E, 6A, 7).

The most immature CMPs were mainly free. Their cytoplasm showed a dual aspect with mesenchymal features, such as free ribosomes and few cisternae of rough endoplasmic reticulum (Fig. 3B), a lacunar aspect very similar to that of the ICLC (Fig. 3A), and cardiac features such as typical cardiac mitochondria (large, with a clear matrix and numerous and long christae and filaments) (Figs. 4, 5). None of them has a complete basal lamina (Fig. 3B).

The lesser immature CMPs were both free (Fig. 4) and grouped in columns of two to three cells (Figs. 6A, 7) and showed many features characteristic for the myocardial lineage cells and identical to those reported for the immature cardiomyocytes of mouse embryos [74]:

1 Bundles of filaments often attached to plasmalemmal dense bands (Fig. 4B, C);
2 Intracytoplasmatic dense bodies that give origin to primordial sarcomeres and primordial Z lines (Figs. 4C, 5E);
3 Numerous caveolae (Fig. 5B);
4 Intracytoplasmatic vesicles often aligned and confluent (to give origin to primordial T tubules) (Fig. 5B);
5 Intracytoplasmatic (Figs. 4C, 5A, C, 6B) and peripheral (Fig. 5B) desmosome-like structures (primordial intercalated discs) sometimes forming a complete intercalated disc (Fig. 7);
6 Subsarcolemmal leptomeric fibrils (Figs. 4C, 5A, D, 7) and
7 A continuous basal lamina (Figs. 4, 6, 7).

The desmosome-like structures often seem to organize thin filaments (Fig. 5A, C) while thick filaments appear to be organized by leptofibrils (Fig. 5A, D). Leptofibrils in CMP showed filamentous bundles periodically crossed (periodicity: 140–160 nm) by three to nine electron dense small bands (about 30 nm wide). The length of leptofibrils varied between 1.03 and 2.21 μm (mean **=** 1.21 μm) and their thickness between 0.16 and 0.83 μm (mean **=** 0.41 μm). Z-like dense structures are visible at the end of leptofibrils (Figs. 4C, 5A, D).

Nerve fibres were frequently seen in the proximity of the CMP columns in contact with either the ICLCs (Figs. 6, 8) or the CMPs (Fig. 12).

### Relationships between ICLCs and CMPs

The ICLCs accompany all the CMPs, strictly apposed to them, independently of their degree of differentiation and mainly oriented parallel to their main axis (Figs. 4A, 6, 7). The mean distance between ICLCs and CMPs was 0.10 **±** 0.05 μm (minimum **=** 0.04 μm; maximum **=** 0.30 μm). Sometimes the ICLCs are directly anchored to the CMPs (Figs. 10, 11) or to their basal lamina (Figs. 6, 8, 10) by means of plasmalemmal areas reinforced by an electron-dense material (Fig. 10). The basal lamina completely surrounds the more mature CMPs (Figs. 4, 6) and when these cells are grouped to form a column this lamina, always lined by the ICLCs, surrounds the entire column often exceeding it at its poles (Figs. 4B, 6, 8, 10). Interestingly, the exceeding basal lamina might continue as a discrete tract, always bordered by the ICLCs (Figs. 4B, 6, 8, 10) and in some images seems to contain an ICLC process (Figs. 7B, 9). The final architecture results in a three-dimensional tunnel-like structure with the CMPs inside, a basal lamina surrounding the column and the ICLCs on the external side of the basal lamina. Primitive cilium, directed to the main axis of the cardiomyoblasts column (Fig. 13), is often present in the ICLCs.

Often, shed microvesicles or exocytotic multi-vesicular bodies (Fig. 11) could be seen in between ICLC and CMP. The microvesicles (80–150 nm diameter) appeared to be generated by ICLC through an exocytotic process as single units or enclosed multi-vesicular bodies (0.5–1 μm wide).

### Cardiac stem cell niches

The CSCNs were located in the subepicardial area adjacent to the coronary arteries at the emerging point from aorta (Figs. 1C, 14) and showed no separation limit from the surrounding interstitium. The CMPs were noticeable in the inner part of the subepicardial space next to the myocardium and they were not seen immediately beneath the mesothelial layer. The extracellular matrix is loose and contains spare bundles of collagen and few elastic fibres.

The cells present in the niche, CMPs, ICLCs, macrophages, mononuclear cells with stem appearance and nerves, showed a tendency to form clusters around capillaries (Fig. 14). Infrequently, CMPs in different stages of development have been seen nearby adult cardiomyocyte and a basal lamina seems to connect each other (Fig. 14C). TEM images showed no cellular connections of the CMPs with other cells except the ICLCs (Figs. 2–4, 6, 7, 13), nerves (Figs. 12, 14) and other CMPs (Figs. 6A, 14C).

## Discussion

This study allowed us to identify different types of cells in the subepicardium of adult mouse. Some of them were present everywhere beneath epicardium, while others were exclusively located nearby the origin of the coronary arteries and aorta in a CSCN. At this location, the cell types identified were cardiomyocytes with immature features (named CMPs), ICLCs, fibroblasts, adipocytes, macrophages, lymphocytes, mononuclear cells, mast cells, free smooth muscle cells, Schwann cells and nerve fibres. The CMPs showed different degrees of immaturity, some of them resembling mesenchymal cells (MSCs) or ICLCs and others mature cardiomyocytes. Interestingly, all of the immature cardiomyocytes have peculiar spatial relationships with the ICLCs.

The CMPs were clearly recognizable as belonging to the cardiomyocyte lineage due to their specific ultrastructural features. Indeed, all of them possess mitochondria with features identical to those of the mature cells and filaments arranged as in the developing cardiomyoblasts [74]. The lesser differentiated CMPs have few cisternae of rough endoplasmic reticulum, a discontinuous basal lamina and bundles of filaments free in the cytoplasm or attached to dense bodies (primordial Z lines). These cells resemble those described in the mouse heart at E9 [74]. The more differentiated cells having a continuous basal lamina, incomplete or complete intercalated discs and primordial sarcomeres resemble the cardiomyoblasts described in the mouse heart at E15 [74]. Moreover, in the cytoplasm of the more mature cells leptomeric fibrils are present [75]. In the adult heart, these structures have been observed associated with structural remodelling, myofibrillar genesis and in the surviving rat cardiomyocytes located in the perinecrotic zone of the infarct [76].

The presence of CMPs in the adult heart represents an important finding and confirms recent reports about the presence of a small pool of cardiac stem and progenitor cells in the adult mammalian myocardium, including mouse [69] and human beings [17]. We have found immature cardiac cells as well as ICLCs in a subepicardial location, this appearing not surprising since it is well known that during embryonic life cardiomyocytes directly origin from epicardium [74], interstitial stromal cells origin from mesenchymal epicardium-derived cells and, indeed, epicardial stem cells isolated and cultured can generate new cardiomyocytes [15, 17]. What we consider of particular importance is that adult mice CMPs are located at a specific site under the epicardium and close to the origin of the coronary arteries and aorta. This finding might help when searching for these cells in order to cultivate or to activate them for heart regeneration.

Recently, very important new insights strongly support the concept that the heart contains the stem cells grouped in niches, i.e.interstitial structures containing either cardio-stem cells or lineage-committed cells connected to supporting cells represented by mature cardiomyocytes and fibroblasts [18, 69]. Therefore, a CSCN requires the co-existence of two different types of cells, stem cells and supporting cells, both of which are necessary to heart renewing and, possibly, repair [68–71]. By junctional and adhesion proteins the cells from these niches are connected structurally and functionally to the pre-existing cardiomyocytes and fibroblasts [69], and the surrounding microenvironment and physical contacts define a niche and the diffusible factors involved in stem cell regulation are crucial for its integrity [70–73, 77].

Since the present study stresses the intercellular connections existing between CMPs and ICLCs, our hypothesis is that taken together these two cell types might be considered forming an actually CSCN. Indeed, the ICLCs accompany the immature cardiac cells all along their length, strictly apposed to them, and are anchored to the basal lamina of the more differentiated cells. When the CMPs are grouped to form a column, the basal lamina, always bordered by the ICLCs, envelops the column and continues at its two poles for a discrete tract. This organization results in a three-dimensional tunnel-like structure made by immature cells inside, a basal lamina around them and ICLCs on the external side. The resulting architecture has an impressive similarity to that found in the adult myocardium [53–59] and gut muscle wall [67]. This architecture is suggestive for an important influence played by the ICLCs on the CMP three-dimensional organization and interconnections and, possibly, also on cardiac repair. In this regard, implantation of MSCs seeded on small intestinal submucosa showed to improve the differentiation of MSCs in cardiomyocytes and smooth muscle cells and the cardiac repair in a rabbit chronic myocardial infarction model [78] thus stressing the importance of MSCs, we presume to be ICLCs in this case [67], in regenerative process. Besides it has been showed that adult serosal mesothelium retains much of its potential of differentiation, not only in the epicardium [79], but also in the gut [80–81], where we suppose that ICLCs [82] are involved.

CMPs are spread out around coronary artery, rather than forming a distinct structure that could be pointed as a niche in the subepicardial area – the cardiac stem cells niche is more likely an open area than an enclosed anatomical structure. This remark points towards the importance of the existence of intercellular communications. As an example, one special intercellular communication between ICLCs and CMPs occurs through shed microvesicles or exosomes (Fig. 11). Recent studies [71, 83–87] reported that microvesicles transfer tissue specific mRNA and proteins and by genetic modulation could determine differentiation and commitment of stem cells. This newly recognized system of intercellular communications seems to have a critical role in physiological and pathological processes and offers a new research direction for stem cell therapy [86, 87]. For all the above reasons we consider the ICLCs network a prerequisite to define an ‘informational space’ proper for cardiac stem cells survival and to control informational ‘packages’ delivered at a distance through microvesicles. Another interesting aspect suggests that ICLCs might be a source of the regenerative cardiomyocytes since (see Fig. 9) ICLC processes might be clearly seen enclosed in the basal lamina of a column. The existence of stromal cells with possible influence on the cardiomyocytes growth and differentiation was already proposed [15, 18, 26, 69, 77] but not yet clarified. A recent study showed that activated CMPs migrate from atrial niches not through coronary circulation but through the interstitium and it was supposed that this migration occurs within tunnels defined by fibronectin [88]. In our opinion, this influence in the adult heart should be the same as that followed during the embryological steps that succeed starting by the trabecular up to the com-pactation of the myocardium. Briefly, we suggest that the ICLCs network is the scaffold capable of guiding the differentiating stem cells whatever their origins are [89–103] and ICLCs are responsible for the secretion of the extracellular matrix molecules functioning as signalling routes necessary to maintain or to repair the myocardial architecture (Figs. 15, 16).

**15:**
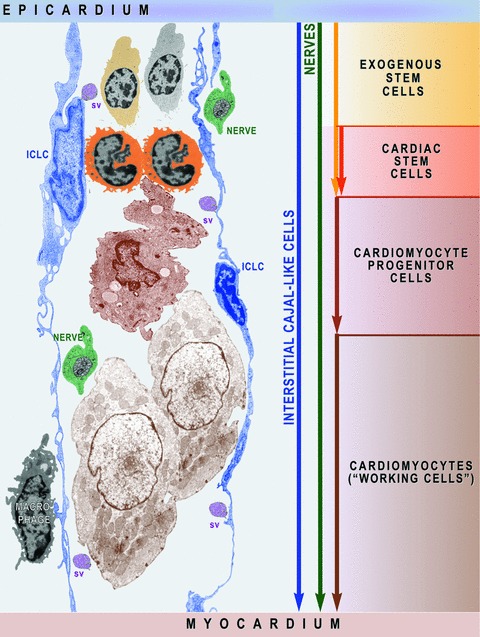
Schematic representation (based on electron micrographs from Figs. 3A inset, 4C, 6A, 12B, 14B) shows that ICLC network is a prerequisite for myocardial cellular homeostasis shaping the scaffold required to activate exogenous and endogenous stem cells and to guide cardiomyocyte progenitors’ migration. Myocardial ICLCs communicate at distance with nurse ICLCs from epicardial niches and deliver information ‘packages’ via shed vesicles (sv). Macrophages and nerves assist the ICLCs to monitor the cardiac renewing.

**16:**
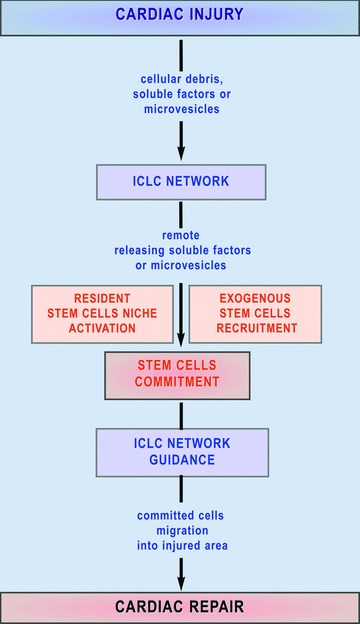
Mechanism proposed for the ICLC involvement in myocardial repair. A cardiac injury could determine a remote cellular reaction in cardiac stem cell niches or could initiate the recruitment of the exogenous adult stem cells via the ICLC wide network. Committed stem cells are guided to migrate into the injured tissue under paracrine signals released by the ICLC.

In summary, we provide here for the first time the ultra-structural description of the CSCNs containing resident CMPs and ICLCs in the adult mice myocardium and prove out of doubt their reality. More than that, the existence in the adult of CMPs in different stages of development proves that cardiac renewing is a continuous process. Here we provide a detailed description of the complex hetero-cellular interactions between CMPs and ICLCs. We can also confirm that the ICLCs make a three-dimensional network in the interstitium of atria, ventricles and epicardium of the adult heart. The CSCNs, that are rare and probably located in strategic areas of the heart, might be integrated in the heart architecture through this wide ICLCs network. A possible reason for an ineffective regeneration of the myocardium in the injuries affecting large areas is that the ICLCs supporting network is broken or the ICLCs have changed their phenotype.

In conclusion, the ICLCs could be not only genuine nurse cells essential for control of stem cells maintenance and lineage commitment, but also be responsible for regulatory mechanisms that control the endogenous or exogenous stem cell activation and a support for the progenitors migration towards a damaged region of the heart. The hope now is to have a successful cell therapy leading the CSCNs to restore the myocardium, and the ICLC, being a key local player, must be taken into consideration in the regenerative cardiology.
